# Perceptions of Intra-Uterine Device Users in Mirebalais, Haiti: A Mixed Methods Study

**DOI:** 10.29024/aogh.2375

**Published:** 2018-11-05

**Authors:** Marie Boller, Meredith Jean-Baptiste, Christophe Millien, Theophile Renise, Marisa Nádas

**Affiliations:** 1 Albert Einstein College of Medicine, Department of Obstetrics and Gynecology and Women’s Health, 1300 Morris Park Avenue, Bronx, New York, US; 2 EqualHealth, Brookline, MA, US; 3 Partners in Health, 800 Boylston Street, Suite 1400, Boston, MA, US; 4 Zanmi Lasante, Port-au-Prince, HT

## Abstract

**Introduction:**

IUDs are safe, effective, and used worldwide to prevent unintended pregnancy. However, uptake in Haiti is low. There are limited data on IUD choice and experience in low resource settings; anecdotal reports from providers in Haiti have suggested that Haitian women are unlikely to choose to use or be satisfied with the IUD. The objective of this study is to explore the perceptions of a cohort of IUD users in Mirebalais, Haiti.

**Methods:**

In June and July 2015, an IRB-approved mixed methods study of women over age eighteen with hormonal or copper IUDs inserted at Hôpital Universitaire de Mirebalais (HUM) was performed in Mirebalais, Haiti.

**Results:**

Twenty-one eligible women participated, out of 58 women identified as eligible. Most women (81%) reported using the copper IUD; most (86%) had used the IUD for 6 months or more. Over half were under 30 years old (62%) and most had completed primary school or less (76%). Almost all (91%) reported prior pregnancies; 65% did not desire more children. The majority of participants were satisfied with the IUD, with 70% being very satisfied and 25% somewhat satisfied. Most women (71%) reported no very bothersome side effects, and would recommend the IUD to others (86%). Qualitative data highlighted positive perceptions of the IUD among users, as well as misperceptions and lack of knowledge regarding the IUD among members of their communities.

**Conclusion/Implications:**

Understanding of culture-specific perceptions is critical in addressing barriers to IUD uptake. Our findings indicate that IUDs can be an acceptable contraceptive method for women in Haiti, and suggest the possibility that increased access to the IUD may lead to increased acceptance of this method.

## Background

Every year, 80 million women worldwide become pregnant without intention. Unintended pregnancy has a particularly devastating impact in developing countries, where approximately 800 women die every day due to complications related to pregnancy. Most of these deaths can be avoided [1].

The global unmet need for family planning remains vast, affecting low and middle-income countries most acutely. Two hundred twenty-two million women in low and middle-income countries who do not desire pregnancy are not using contraception [1]. This unmet need drives the mission of Family Planning 2020 and other global initiatives designed to empower women and girls to make choices regarding family planning. With access to effective contraception, women are empowered to achieve higher educational levels and build more financially secure families – promoting the health of communities and enhancing the strength of national economies. While efforts to expand access to modern methods of contraception in low-resource settings gain momentum, it is critical that they be informed and targeted to population needs and individual preferences.

Globally, the prevalence of modern contraceptive use was 57% in 2015 [2]. In Haiti, uptake lags significantly at 31% [3]. In 2014, approximately 656,000 Haitian women desiring contraception were not using a reliable method [4].

The IUD is used by 14% of reproductive age women worldwide, and is the most commonly used long-acting reversible contraception (LARC) [2]. The IUD enables women to delay childbearing by up to 10 years in the case of the copper IUD, and five years in the case of the hormonal IUD. It is over 99% effective, carries a low risk of adverse events, and does not require user adherence to be effective [5]. However, this method continues to be underutilized in developing countries [6]. In Haiti, IUD uptake among reproductive age women is reported to be 0.1% in the 2012 Enquête Mortalité, Morbidité et Utilisation des Services (EMMUS-V) report [3] and 0.08% in the 2013 Ministère de la Santé Publique et de la Population (MSPP) report [7].

Limited data exists on IUD choice and experience in low-resource settings. Studies indicate that low IUD utilization may be largely influenced by misperceptions among women [8]. Common perceptions that influence a woman’s decision to avoid using or discontinue using the IUD include concerns of feeling a foreign object inside the uterus, concern about alterations in the bleeding and menstrual cycle, worry that a partner would complain about feeling the IUD during intercourse, and fear that the IUD would cause cancer or sterility [8,9,10].

To our knowledge, no research has previously been conducted on perceptions of the IUD in Haiti, but anecdotal reports suggest that Haitian women are unlikely to choose or be satisfied with the IUD and that providers also have negative perceptions of this method.

In November 2013, an IUD counseling and insertion training was conducted for women’s health providers at Hôpital Universitaire de Mirebalais (HUM), in Mirebalais, Haiti. Concurrently, a supply of IUDs was obtained through donation and UNFPA sourcing. IUD counseling was incorporated into daily standard group family planning counseling, and IUD uptake began. With this study, we describe and quantify the experience of a cohort of Haitian women who had an IUD inserted at HUM. Our primary objectives are to: a) describe user experience, including side effects and degree of satisfaction; and b) identify the factors that influence use or discontinuation of the IUD in this context. The secondary objective of our study is to learn from Haitian IUD users what women in their communities are saying about the IUD.

## Methods

A mixed-methods study with purposive sampling was performed in Mirebalais, Haiti in June and July 2015 through individual interviews. A quantitative component was necessary in order to determine patterns of perceptions. A qualitative component was added, consisting of three open-ended questions included in the interview tool, in order to elicit unique and nuanced opinions about the IUD. Inclusion criteria for study participation were age of over 18 and having had a copper or hormonal IUD inserted at HUM between November 1, 2013 and July 12, 2015. Exclusion criterion was ages less than 18 years. Family planning clinic records were used to identify potential study participants, and all women who met the criteria were reached out to by phone and invited to participate in the study. Participants traveled to HUM to be interviewed. Participant travel costs were reimbursed, and a small meal was provided.

Trained interviewers administered a structured interview consisting of 26 closed-ended questions and three open-ended questions with each participant. Approximate time to completion of the interview was 20 minutes. Interviewers were members of the research team and were trained in interview technique by the principal investigator. All interviewers were fluent in Haitian Kreyol. As interviews were conducted, participant responses were recorded by hand in Haitian Kreyol by the interviewer. Closed-ended questions addressed IUD users’ experiences with the IUD, including insertion experience, side effects and degree of satisfaction. Satisfaction was measured by a question asking IUD users to rate their satisfaction on a scale from “very dissatisfied” to “very satisfied.” Perceptions were further addressed in three open-ended questions. The interview tool was designed by the authors of this study and was not a validated tool; however, it was informed by other published studies on IUD experience (see Appendix A).

Responses were transferred to an electronic format and translated into English by the bilingual research team for analysis. Descriptive analysis of quantitative data was performed using Microsoft Excel. Answers to open-ended questions were analyzed by hand for themes. As this was a pilot study with a maximum study population of the number of IUD users from HUM since initiation of the program, results were not powered for statistical significance.

There was no identifying information obtained from participants as part of this study. Verbal consent was obtained from each participant and documented by an interviewer’s signature prior to the interview. Ethics approval to carry out this research was obtained from the IRB of Albert Einstein College of Medicine and the IRB of Zanmi Lasante.

## Results

Fifty-eight women were identified as eligible through review of HUM family planning clinic records and telephone contact was attempted by study staff. Of the 26 women successfully contacted by phone, all agreed to participate in the study; of those, 22 completed the study. One woman was found to be ineligible following completion of the study because her IUD was not inserted at HUM; data from her interview was removed from the study results. Four women did not show for the interview. Demographic characteristics of participants are shown in Table 1. Participants listed multiple responses when appropriate.

**Table 1:** Participant Demographics.

		Total respondents (n = 21)	

**Age (years)**	<25	6	28.6%
	25–29	7	33.3%
	30–34	4	19.0%
	35–39	2	9.5%
	>39	2	9.5%
**Residence**	Town	12	57.1%
	Countryside	9	42.9%
**Number of times pregnant**	0	2	9.5%
	1	4	19.0%
	2	5	23.8%
	3	6	28.6%
	4+	4	19.0%
**Number of living children**	0	2	9.5%
	1	5	23.8%
	2	8	38.1%
	3	4	19.0%
	4+	2	9.5%
**Age of youngest child (years):**	<1	2	10.5
	1–4	16	84.2
	>4	1	5.3
**Highest level of education**	No formal education	1	4.8%
	Did not complete primary education	7	33.3
	Completed primary education	8	38.1
	Completed secondary education	3	14.3
	Completed university	2	9.5%
**Methods of contraception used before IUD**	None	5	23.8
	Birth control pills	4	19.0%
	Injectable contraception	12	57.1
	Condoms	4	19.0%
	Implant	0	0.0%
	Natural family planning (incl. breastfeeding)	3	14.3
	Tubal ligation	1	4.8%
	Other	2	9.5%
**Sexual partner at time of IUD placement**	Yes	21	100.00
	No	0	0%
**Living with partner at time of IUD placement**	Yes	15	71.4
	No	2	9.5%
	No response	4	19.0%
**Discussed decision to get IUD with partner**	Yes	15	71.4
	No	4	19.0%
	No response	2	9.5%
**Partner in agreement with decision**	Yes	15	71.4
	No	1	4.8%
	No response	5	23.8%
**Would like to have more children**	Yes	4	20.0
	No	13	65.0%
	Don’t know	3	15.0%
**Duration of IUD use (months)**	<6	2	9.5
	6–12	10	47.6%
	>12	8	38.1%
	not answered	1	4.8%
**Type of IUD inserted**	Hormonal	3	14.3%
	Copper	17	81.0%
	Unknown	1	4.8%

The majority of participants were satisfied with the IUD, with 70% being very satisfied and 25% somewhat satisfied. Most women (71%) reported no significant bothersome side effects and would recommend the IUD to others (86%). Table 2 shows participant satisfaction rates. Tables 3 and 4 show reported side effects and reasons cited for choosing the IUD, respectively. Participants selected all applicable responses.

**Table 2:** Reported Experience with IUD.

		n=21	

**Degree of Satisfaction with IUD**	Very satisfied	14	70.0%
	Somewhat satisfied	5	25.0%
	Somewhat dissatisfied	0	0.0%
	Very dissatisfied	1	5.0%
**Degree of pain during insertion**	Not painful	10	47.6%
	Somewhat painful	7	33.3%
	Very painful	4	19.0%
**Partner complained about strings**	Yes	5	23.8
	No	16	76.2%
**Found any side effects to be very bothersome**	Yes	6	28.6
	No	15	71.4%

**Table 3:** Reported Side Effects.

	(n = 21)	

**Change in vaginal discharge/vaginal infection**	10	47.6%
**Heavier menstrual flow**	6	28.6%
**Cramping – minimal**	5	23.8%
**Pain during intercourse**	4	19.0%
**Feeling tired all the time**	4	19.0%
**Heavy irregular bleeding**	3	14.3%
**None**	2	9.5%
**Dizziness**	2	9.5%
**Light irregular bleeding**	2	9.5%
**Cramping – severe**	1	4.8%
**Back pain**	1	4.8%

**Table 4:** Reasons for Choosing IUD.

	(n = 21)	

**Works for a long time**	11	52.4%
**Prefer to use it over other forms of family planning**	9	42.9%
**Nurse, midwife, or doctor recommendation**	8	38.1%
**I will see my period regularly (copper IUD)**	4	19.0%
**Easy to use**	3	14.3%
**I don’t want to have more children**	3	14.3%
**Effective (works well)**	2	9.5%
**Less side effects**	2	9.5%
**No hormones (copper IUD)**	2	9.5%
**It will stop my periods (amenorrhea with Progestin IUD)**	1	4.8%
**Family or friend recommendation**	1	4.8%
**“More trust, I can’t forget appointment for the method”**	1	4.8%
**“Medical reason”**	1	4.8%

Participants who responded “yes” to the question “Would you recommend the IUD to others?” were asked why. In addition, participants were asked, “What do you tell people about the IUD?” Responses to both questions covered similar themes. The majority of participants made positive statements regarding the IUD. Twelve of the 21 participants used the word “good,” “better,” or “best” to describe the IUD; the most repeated phrase in the responses was “it is a good method that lasts a long time.” In discussing why they would recommend the IUD to others, two participants cited the dependability of the IUD and three cited the lack of hormones and/or side effects. Three cited the effectiveness of the IUD, with one stating that she would recommend the IUD to others “to limit the power to have children.” Another participant stated, “My friends ask me because I am not pregnant often, so I explain to them the type of contraception I use.”

Fifteen (71%) of the participants reported that what they tell others about the IUD is positive. These responses included:

“It’s a method that is very effective, easy to use, and does not have too many complications.”

“They can trust it because I had it put in and it’s good. When I had it placed, I was scared that I would have pain when I had sex, but I never had pain and my partner never complained of it.”

“I tell them not to be afraid.”

In response to the question “What do you tell people about the IUD?” five participants gave equivocal responses, stating that they did not discuss the IUD with others. One stated, “I don’t say anything. When I talk about contraception, I say I am going to remove it to not use contraception for such a long time.” Another stated, “[I tell them] I am going to the hospital to get advice from the nurses and doctors.” One response to this question was negative regarding the IUD, stating, “The method is normal. Only thing is the side effects. It makes me sleepy and makes my waist hurt.”

Of the five participants who gave equivocal responses to the question above, three said that they would not recommend the IUD to others. One did not give a reason, another stated, “I am not yet used to it.” The third stated, “They do not have the same problem as me, they don’t know the IUD exists.”

One participant reported discontinuing the IUD, reporting that the IUD had given her an infection. Due to the small sample size, we were not able to gather any meaningful data on why women choose to discontinue the IUD method.

One third of women reported that they had felt pressured by others to have their IUD removed. The secondary objective of our study – to find out what other people in the community were saying about the IUD – was addressed by an open-ended question. Eight participants gave responses suggesting lack of knowledge of the IUD in their communities. These included: “They don’t know it,” “It is not a method they know too well in my community,” and “They don’t say anything. They know this method but they don’t use it.” One participant suggested a lack of access in her community, stating, “They can’t travel to get here.”

Three participants gave positive responses: “It’s good,” and “They use it and they don’t say anything. There are women who use the IUD and I don’t hear any criticism of it.” The remaining nine (43%) of the participant responses described negative perceptions of the IUD in their communities. A selection is included below:

“They don’t want to use other methods they use parsley.”

“They tell me I’m too young, the method is too long, it might not be good for me”

“It can kill me, it can make me unable to have children, it can destroy the uterus.”

“They are scared to use it due to bleeding problems and for the strings because they think they can be annoying and also they are nervous about using a method for such a long time.”

“It causes uterine cancer and infections.”

“People don’t know it. I usually speak to them about it. The women are afraid because of where it’s located.”

## Discussion

This study is, to our knowledge, the first to explore perceptions of IUD users in Haiti, a country with a large unmet need for contraception and yet with one of the lowest rates of IUD uptake in the world. Limitations of this study include the sample size, necessitated by the small population of eligible participants, as well as challenges with contacting eligible women. Incentive to participate was limited to travel reimbursement and provision of a small meal to avoid undue influence that could compromise the informed consent process. The dual role of investigators as clinical staff at HUM involved in delivery of care to study participants is a potential source of bias, possibly impacting the comfort level of the participants to share negative experiences with the IUD. Recall bias of participants may also have impacted the quality of the data; however, the potential for recall bias was minimized by recruitment of patients who had started using the IUD within the previous two years, with approximately 50% having had the IUD inserted 6–12 months prior to the study. We were unable to evaluate reasons for discontinuing the IUD due to inadequate sample size, and further research is needed in Haiti to describe factors that affect women’s decision to discontinue using the IUD.

The results of this pilot study indicate a high level of satisfaction among women who chose to have an IUD placed at HUM. In addition, this study provides insight into perceptions of the IUD within communities surrounding Mirebalais. Although 86% of the women in this study reported that they would recommend the IUD to others, almost half of the women in our study reported negative perceptions of the IUD in their community. This information can be used to inform individualized and population-based education and contraceptive counseling.

Four women in the study stated that they do not discuss their IUD with others. Others reported that women in their families or communities do not discuss the IUD, with one stating, “They don’t say anything. They know this method but they don’t use it.” These responses suggest the possibility that discussing the IUD or contraception in general may be a taboo subject among some women in Haiti, which is a question for further study.

Our study findings are consistent with other studies describing high satisfaction of IUD users in diverse settings and communities. Although generalizability is limited by small sample size, these results suggest that acceptability of the IUD to Haitian women is comparable to other communities around the world.

## Conclusion

Our results indicate that IUDs can be compatible with Haitian culture, contrary to widespread belief that IUDs will never be accepted in Haiti. These findings reflect the responses of a pilot cohort of women, some of the first in Haiti to be offered the IUD. Given the uptake of IUDs at HUM and the high rate of satisfaction with the method, we posit that low uptake of the IUD can be more likely attributed to limited availability of IUDs and contraceptive counseling that encompasses the IUD, rather than rejection of the method by Haitian women. Our results suggest that increased access to long-acting reversible contraceptive options such as the IUD will lead to increased acceptance of these methods, as much through the gradual process of word-of-mouth between women as through formal programming. We believe that our study results can inform future family planning programming and enable Haitian providers to offer IUD counseling and services adapted to the Haitian context, ultimately leading to more informed contraceptive decision-making by Haitian women.

It is our hope that increased uptake of the IUD will promote empowerment of Haitian women to choose the families they build and enable them to secure more stable financial futures, making Haiti stronger as a nation.

## Additional File

The additional file for this article can be found as follows:

10.29024/aogh.2375.s1 Appendix A. Interview Tool.

## Figures and Tables

**Table:** 

**HUM**	Hôpital Universitaire de Mirebalais
**IUD**	Intra-Uterine Device
**LARC**	Long-Acting Reversible Contraception
**UNFPA**	United Nations Population Fund
