# Supplementary material for: Perceptions of Intra-Uterine Device Users in Mirebalais, Haiti: A Mixed Methods Study
Source: Ann Glob Health. 2018 Nov 5;84(4):663–9. doi: 10.29024/aogh.2375 (PMC6748221; doi:10.29024/aogh.2375)
Supplement: Appendix A. — Interview Tool. [file agh-84-4-2375-s1.pdf]

User Perceptions of the IUD in Mirebalais, Haiti
Patient Interview
Date of interview:_________________

1. How old are you? ______

2. Where is your residence?
    a. In town
    b. In the countryside

3. G ___ P ___ A ___ EV ___

4. How old is your youngest child? ______

5. What is the highest level of education you have attained? *(Read all answers)*
    a. No formal education obtained
    b. Did not complete primary education
    c. Completed primary education
    d. Completed secondary education
    e. Completed university

6. Before the IUD, what other methods of contraception had you used in the past:
    a. None
    b. Birth control pills
    c. Injectable contraception
    d. Condoms
    e. Implant
    f. Natural Family Planning (including breastfeeding)
    g. Other: ____________________________

7. Did you have a sexual partner at the time you decided to have the IUD placed?
    a. Yes
    b. No

If NO, skip to question 11

8. Were you living with your partner at the time you decided to have the IUD placed?
    a. Yes
    b. No

9. Did you discuss your decision to get an IUD with your partner?
    a. Yes
    b. No

10. Did your partner agree with your decision?
    a. Yes
    b. No

11. Would you like to have more children?
    a. Yes
    b. No

12. Do you still have the IUD?
    a. Yes
    b. No

13. How long have you had or did you have the IUD? _______

14. If you had the IUD removed, why did you have it removed? *(Read all answers)*
    a. Pain
    b. Heavy bleeding
    c. Irregular bleeding
    d. Desire for pregnancy
    e. Someone told me that I should have it removed
    f. Other: ____

If IUD was REMOVED, skip to question 17

15. How long are you planning to keep the IUD in? _______

16. How satisfied are you with the IUD? *(Read all answers)*
    a. Very satisfied
    b. Somewhat satisfied
    c. Somewhat dissatisfied
    d. Very dissatisfied

17. Which IUD do you have / did you have? *(Read all answers)*
    a. Hormonal (5 year)
    b. Copper (12 year)

18. What aspects of the IUD were most important to you when you chose it? *(Read all answers, select up to three)*
    a. Easy to use
    b. Works for a long time (5 or 12 years)
    c. Effective (works well)
    d. It will stop my periods (amenorrhea with progestin IUD)
    e. I will see my period regularly (copper IUD)
    f. No hormones (copper IUD)
    g. Nurse, midwife, or doctor recommendation
    h. Family or friend recommendation

i. Prefer to use it over other forms of family planning (ie: condom, birth control pill)
j. Cost
k. Other: ____

19. How would you rate the degree of pain you felt during the insertion? *(Read all answers)*
    a. Not painful
    b. Somewhat painful
    c. Very painful

20. What side effects have you experienced? *(Read all and select all that apply)*
    a. None
    b. No bleeding—not even a menstrual period
    c. Heavier menstrual flow
    d. Cramping—minimal
    e. Cramping—severe
    f. Light irregular bleeding
    g. Heavy irregular bleeding
    h. Change in vaginal discharge/vaginal infection
    i. Back pain
    j. Pain during intercourse
    k. Feeling tired all the time
    l. Dizziness
    m. Other: ____

21. Has your partner complained about the strings post-insertion?
    a. Yes
    b. No

22. Have you found any side effects to be very bothersome?
    a. Yes
    b. No

23. If yes, which ones? *(Read all answers and select all that apply)*
    a. No bleeding—not even a menstrual period
    b. Heavier menstrual flow
    c. Cramping—minimal
    d. Cramping—severe
    e. Light irregular bleeding
    f. Heavy irregular bleeding
    g. Change in vaginal discharge/vaginal infection
    h. Back pain
    i. Pain during intercourse
    j. Feeling tired all the time
    k. Dizziness

l. Other: ____________________________________

24. Have you felt pressure from others to remove the IUD?
    a. Yes
    b. No

25. If yes, from whom? _______________

26. Would you recommend the IUD to other women?
    a. Yes
    b. No

27. Why?

28. What are other women in your family or community saying about the IUD?

29. What do you tell people about the IUD?

Thank you so much for taking the time to speak with us today. Your answers will help improve family planning options for women in Haiti. Do you have any questions for me?
